# Kirner’s deformity of the fifth finger: a case report

**DOI:** 10.1186/s12891-016-1300-8

**Published:** 2016-10-21

**Authors:** Melkamu Adeb, Daichi Hayashi, Yogesh Kumar

**Affiliations:** 1 Department of Radiology, Bridgeport Hospital, Yale New Haven Health, 267 Grant Street, Bridgeport, CT 06610 USA; 2 Department of Radiology, Boston University School of Medicine, Boston, MA USA

**Keywords:** Kirner’s, Deformity, Dystelephalangy, Case report

## Abstract

**Background:**

Kirner’s deformity is a rare bony deformity that is characterized by radial and volar curvature of the distal phalanx of the fifth finger. Affected patients usually present after the age of 5 years, with girls more affected than boys and bilateral involvement more common than unilateral.

**Case presentation:**

We report a case of an eight-year-old girl who presented with progressive deformity of the right little finger. Radiographic evaluation revealed volar and radial curvature of the distal phalanx of the right fifth digit. Magnetic resonance imaging (MRI) further revealed the deformity along with widening of the physeal plate, lack of soft tissue enhancement and normal insertion of the flexor digitorum profundus tendon. The patient was followed conservatively for two years and is now being considered for corrective osteotomy.

**Conclusion:**

Kirner’s deformity is a rare abnormality of unknown etiology. Diagnosis is made with clinical examination and imaging evaluation. Clinicians should be aware of this uncommon deformity and differentiate it from other mimickers such as infection, physeal fracture, camptodactyly, and clinodactyly.

## Background

Kirner’s deformity or dystelephalangy is a rare bony deformity that is characterized by radial and volar deviation of the distal phalanx of the fifth finger [1–8]. The condition is painless but patients may have swelling of the distal interphalangeal (DIP) joint and the development of watch-glass nail in the fingers involved [2]. Most patients present clinically after the age of 5 years (range: 8–14). Earlier onset of the deformity has been described in the extremely rare polytopic form [3]. The frequency of Kirner’s deformity is difficult to ascertain, with reported rates of 0.25 % in the English and 0.15 % in the Japanese series [3, 4]. Girls are affected more frequently than boys. The incidence of this anomaly in fingers other than the little finger is extremely rare [3]. Bilateral and symmetrical involvement is common, with the right hand being more severely involved than the left [4, 5]. In this case report, we discuss the clinical and imaging findings in an eight-year-old girl who presented with classic features of Kirner’s deformity.

## Case presentation

**Fig. 1:**
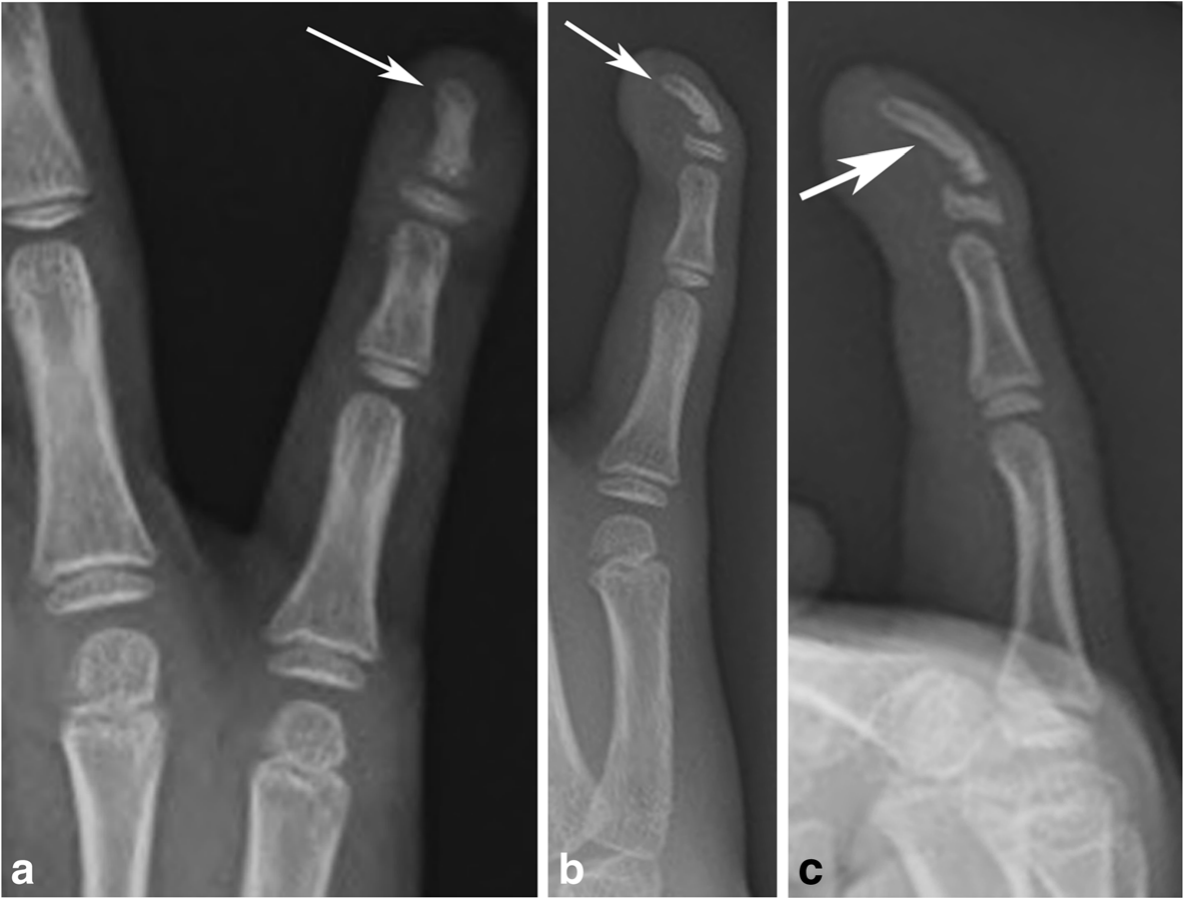
Frontal (**a**), oblique (**b**) and lateral (**c**) radiographs of the right hand show characteristic curvature of the distal phalanx of the fifth digit in palmar and radial directions (*arrows*)

**Fig. 2:**
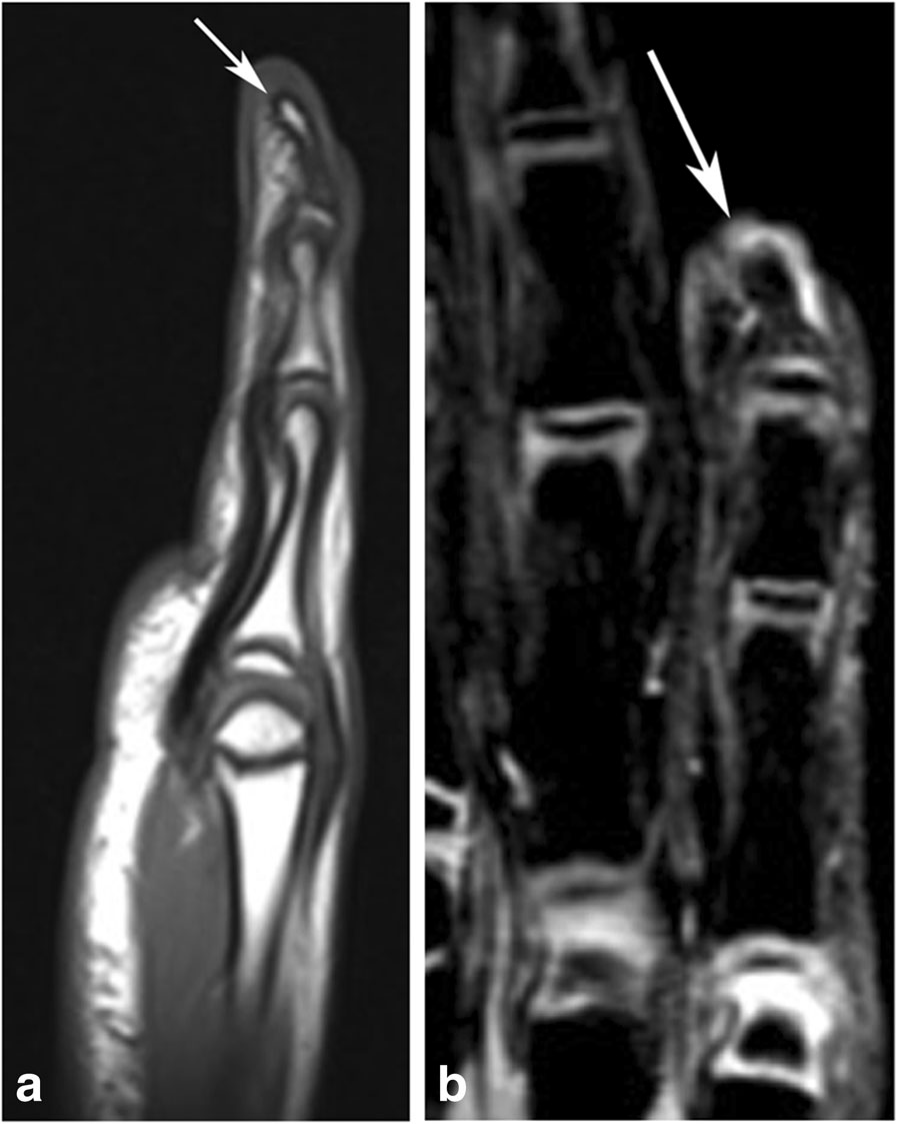
Sagittal T1-weighted (**a**) and coronal gradient recalled echo (**b**) images of the right hand also demonstrate the palmer and radial curvature of the distal phalanx of the fifth digit (*arrows*)

An eight-year-old girl was brought to our hospital by her parents because of progressive deformity of the right fifth finger. Her mother reported to have noted progressive volar curvature of the distal aspect of the right fifth finger over a 3 to 4 months period. There was no history of injury, laceration or trauma of any sort. The patient had no fevers or erythema about the finger. Physical examination revealed extensor lag of the DIP joint and volar curvature of the distal phalanx consistent with a mallet finger. Initial radiographs revealed abnormal volar and radial curvature of the distal phalanx of the right fifth finger with widening of the epiphyseal plate (Fig. 1). Initial differential considerations included a Salter Harris fracture, osteomyelitis and a congenital deformity such as Kirner’s deformity. However, in the absence of trauma and clinical signs and symptoms of infection, possibilities of fracture and infection were considered to be less likely. Magnetic resonance imaging (MRI) was performed to further evaluate the deformity and exclude the possibility of infection. MR images (Fig. 2) demonstrated the abnormal curvatures of the distal phalanx without evidence for underlying osteomyelitis. No abnormal soft tissue enhancement was seen. There was normal insertion of the flexor digitorum profundus (FDP) tendon (Fig. 2), contrary to the findings on some case reports in the literature. The patient was then treated conservatively, with no significant change in the deformity on follow up evaluations. The patient is currently being considered for possible corrective osteotomy.

## Discussion

Kirner’s deformity is a rare bony deformity that is characterized by radial and volar deviation of the distal phalanx of the fifth finger [1–8]. The etiology of this deformity remains unknown. Possible suggested mechanisms include: osteochondritis, osteomalacic changes, aseptic necrosis affecting the epiphysis and not the diaphysis, epiphysiolysis such as in slipped capital femoral epiphysis and abnormal distal insertion of the FDP tendon along the volar surface of the distal phalanx of the fifth finger [1, 6]. However, other studies have shown cases of Kirner’s deformity with normal FDP tendon insertion [1, 6]. Fairbank et al. suggested volar extension of the physis of the distal phalanx as a possible cause resulting in a ‘staple’ like action on the volar aspect of the bone thereby causing volar and radial curvature of the distal phalanx, with reduced growth on the volar side of the distal phalanx [1]. While growth of the cartilage in abnormal dimensions is the likely cause of the ultimate deformity, ranges of deforming forces likely also contribute, including the FDP tendon, extensor mechanism and retinacular ligaments. It is possible that the cartilaginous extension of the physis thus represents a ‘volar bracketed epiphysis’, with an L-shaped rather than the normal C-shaped physis [1]. Kirner’s deformity may be inherited as an autosomal dominant trait with incomplete penetrance, or it may occur sporadically [3, 7]. The patient in our case report had normal insertion of the FDP tendon as can be seen on the MRI study (Fig. 2). However, radiographic evaluation of the patient (Fig. 1) demonstrates widening of the physis in the volar aspect of the distal phalanx supporting the proposed mechanism of volar extension of the physeal plate as a possible cause of Kirner’s deformity.

Radiographic evaluation of Kirner’s deformity reveals various degrees of radial flexion deformities and volar flexion of the distal phalanges, ranging from 5 to 50° [3, 6]. The deviation involves the diaphysis with preservation of the epiphyseal, metaphyseal and articular alignment. The physeal plate tends to be widened, and the diaphysis is sharply narrowed with a loss of normal trabecular bone structure [3, 6]. These radiographic findings are well noted in our case (Fig. 1). Associated subluxation at the DIP may be seen in some cases. The bowing site of Kirner’s deformity depends on the age of the patient: the epiphyseal line in juveniles, the diaphysis in juveniles or adolescents, and the distal tip in adults [3, 6]. MRI may reveal additional findings along with detailed evaluation of the deformity, which include: Abnormalities of the Physeal cartilage, abnormal insertion of the FDP tendon, and the presence or the absence of soft tissue enhancement [2, 4]. In one report, contrast enhancement of the soft tissue in the distal fingers was noted on MRI, possibly signifying the presence of chronic inflammatory process or altered vascularization of the soft tissue of the distal fingers [2]. However, Miller et al suggested this finding to be artifactual, likely related to poor fat suppression while imaging small body parts [8]. MRI evaluation of our patient also did not reveal any abnormal soft tissue contrast enhancement.

Possible differential considerations for Kirner’s deformity include frostbite, physeal fracture and infection [5]. Frostbite typically also involves other fingers, while trauma and infection can usually be excluded by clinical history. Other angular deformities could be in the differential diagnosis including “camptodactyly” in which there is volar flexion deformity at the PIP or DIP joints, or “clinodactyly” which represents radial or ulnar curvature, usually at the fifth DIP joint, as a result of abnormal angulation of the middle phalangeal head [8].

Kirner’s deformity results in little functional limitation of the finger. Available treatment options include: observation, splinting and osteotomy, distraction lengthening, hemiepiphysiodesis, and distal detachment of the FDP tendon. No spontaneous resolution of the deformity has been reported [5, 7]. The indication for surgery is primarily restoration of finger appearance. Mild to moderate deformity can be simply monitored or splinted to retard progression while severe deformity, particularly in a skeletally mature patient, may require one or more osteotomies of the terminal phalanx and, rarely, an amputation [5–7].

## Conclusion

Kirner’s deformity is a rare abnormality characterized by radial and volar deviation of the distal phalanx of the fifth finger. Diagnosis is made with physical examination and imaging evaluation. Radiographs demonstrate varying degrees of volar and radial flexion deformities involving the diaphysis of the distal phalanx. MRI provides additional evaluation of the deformity as well as associated findings such as abnormalities of physeal cartilage, insertion of the FDP tendon and the presence or absence of soft tissue enhancement. Awareness of this uncommon deformity will help pediatricians, orthopedic surgeons, and radiologists to differentiate it from other mimickers such as infection, physeal fracture, camptodactyly, and clinodactyly.

## Abbreviations

DIP: Distal interphalengeal
FDP: Flexor digitorum profundus
MRI: Magnetic resonance imaging
